# Risk factors and a simple model for predicting bile leakage after radical hepatectomy in patients with hepatic alveolar echinococcosis

**DOI:** 10.1097/MD.0000000000008774

**Published:** 2017-11-17

**Authors:** Xianwei Yang, Yiwen Qiu, Wentao Wang, Xi Feng, Shu Shen, Bo Li, Tianfu Wen, Jiayin Yang, Mingqing Xu, Zheyu Chen, Lunan Yan

**Affiliations:** Department of Liver Surgery, West China Hospital of Sichuan University, Chengdu, P.R. China.

**Keywords:** bile leakage, hepatic alveolar echinococcosis, radical hepatectomy, risk factors

## Abstract

Postoperative bile leakage (BL) is a major complication of hepatic alveolar echinococcosis (HAE). The purpose of this study was to identify the risk factors for BL and to establish a simple scoring system for predicting BL.

A total of 152 patients with HAE were included in the study between May 2004 and December 2016. The patient's baseline data, laboratory blood tests, imaging features, and surgical management were collected. Univariate and multivariate analyses were used to screen for factors to predict BL. The cutoff values for those factors and predictive value of a model were determined by receiver operative characteristic curve (ROC) analysis.

BL was detected in 22 of the 152 patients. Univariate analyses showed significant differences in the lesion diameter, levels of lactate dehydrogenase (LDH), alkaline phosphatase, aspartate aminotransferase, alanine aminotransferase and direct bilirubin (DBIL), inferior vena cava invasion, surface area of hepatectomy, blood loss and history of percutaneous transhepatic cholangial drainage between patients with and without BL. On multivariate analyses, DBIL > 7.1 μmol/L, LDH > 194 U/L, lesion diameter > 12 cm and a larger surface area of hepatectomy were independent predictors of BL. The resulting area under the ROC of the scoring model was 0.724 (95% CI, 0.646–0.793).

The lesion diameter, DBIL, larger surface area of hepatectomy, and elevated LDH were the important factors affecting the occurrence of BL after surgery. The risk score model will help the clinician to assess BL before surgery. More studies are needed to confirm the scoring model and risk factors.

## 1 Introduction

Alveolar echinococcosis (AE) is a deadly parasitic disease characterized by tumor-like growth. In addition to endemic areas, AE patients are increasing in Europe, Australia, and the United States, largely due to travel and the domestic dogs.^[1]^ The eggs of *Echinococcus multilocularis* are parasitic on canine intestinal villi, and adults discharge larvae with the feces; these larvae adhere to dog hair or wool and can be released into water or food. Humans are accidentally infected by contact and need only ingest 6 larvae to become infected; these larvae penetrate through the gastrointestinal wall into the portal vein; most stay in the liver, while a few escape to the lungs and other organs.^[2]^ Nearly 70% of hepatic alveolar echinococcosis (HAE) lesions are in the right lobe, and 40% involve the hepatic hilus.^[3]^ Patients do not show obvious early clinical symptoms, manifested as upper abdominal pain, weight loss, and fatigue. Late clinical symptoms may manifest as abdominal distension, fever, and cholestatic jaundice in the advanced stage.

Radical resection is the most effective approach to treat HAE. The 10-year mortality rate for untreated HAE patients is 95%.^[4]^ Only early diagnosis and early treatment can improve the survival rate of HAE patients and the rate of curative resection, confirmed based on the detailed clinical presentation, epidemiological evidence, radiological examination, nucleic acid detection, and serologic tests. Complications such as bile leakage (BL), portal hypertension, Budd–Chiari syndrome and liver rupture have been reported in advanced stages, and these indicate the invasive growth performance of the HAE.^[5]^ Various complications occur after liver resection, but biliary leakage has been one of the most common and complex complications with a revealed incidence ranging from 3.6% to 15.6%^[6–9]^; after hepaticojejunostomy, it ranges from 0.4% to 8%.^[10]^ BL, as a significant complication after hepatectomy,^[6]^ always leads to delayed pull-out of the abdominal drainage tube, prolonged hospital stay, application of additional inspection methods (minimally invasive and endoscopic methods) and interventions, even surgery again.^[7]^ In serious cases, BL can cause abdominal abscess and severe biliary tract inflammation and may result in patient death. BL may be the source of the cut surface of the liver section, small bile duct injury, or anatomical changes after biliary enteric anastomosis. Although various methods have been reported to decrease the risk of BL, such as intraoperative cholangiography,^[11]^ BL tests,^[12]^ and the use of fibrin glue to the cut surface of the liver,^[13]^ BL cannot be completely avoided.^[14]^ Graeter et al^[15]^ reported the hepatobiliary complications diagnosed in 35 (9.8%) patients who had undergone endoscopic interventions. Frei et al^[16]^ reported a late biliary complication rate of 28% in patients with nonresectable HAE. On the other hand, regarding the cystic hydatid disease of BL, the BL rate was 10%^[17]^ to 23.5%.^[18]^

However, many HAE patients with BL after radical hepatectomy have not been reported. There is poor information concerning the risk factors and result of patients with BL. Given the high incidence of BL after hepatectomy with HAE patients, how to precisely estimate the hazards of BL remains vague. Consequently, the aim of this study was to establish a predictive risk scoring system for BL considering the risk factors after hepatectomy with HAE.

## 2 Methods

### 2.1 Patients

We enrolled retrospectively 152 patients who were treated with radical hepatectomy for hepatic AE from the Echinococcus Multilocularis Data Bank and electronic medical records at the West China Hospital of Sichuan University from May 2004 to December 2016. The West China Hospital Research Ethics Committee approved the retrospective analysis of anonymous data involved in this study. Patient data such as gender, age, body mass index (BMI), medical history of operation, abdominal imaging lesion characteristics (diameter and number of lesions, the presence or absence of the vessels and bile ducts involved with the disease), blood loss, preoperative laboratory values (blood cell count, alanine transaminase (ALT), aspartate aminotransferase (AST), γ-glutamyl transpeptidase (GGT), alkaline phosphatase (ALP), and bilirubin), and postoperative complications were analyzed. At least 1 imaging tool of enhanced computed tomography (CT) or magnetic resonance imaging (MRI) for all patients was performed before surgery, and the imaging evaluation included the HAE lesion size, number, extrahepatic disease, vascular invasion, and so on. Percutaneous transhepatic cholangial drainage (PTCD) or endoscopic drainage was performed prior before surgery for patients with jaundice or biliary obstruction. BL was caused by pancreas and duodenum surgery, extrahepatic bile duct resection and reconstruction, which were not included in the study. The patients were at least followed up to postoperative 3 months through liver function tests and physical examinations, and imaging tools with CT and/or ultrasound (US) were performed once every 2 months. The PNM staging system (where the initials P, N and M refer to parasite in the liver, extension to neighboring organs and distant metastasis, respectively) for HAE was developed by the World Health Organization (WHO).^[19]^ According to the presence or absence of main vessels and/or bile ducts invasion in the PNM stage, the patients were divided into early and advanced groups. Early referred to levels I and II, and advanced referred to levels III and IV.

### 2.2 Definitions of bile leakage

BL for this study was defined as proposed by the International Study Group of Liver Surgery (ISGLS),^[20]^ that is, drainage of fluid with a bilirubin level 3 times greater than the serum level on or after postoperative day 3 or the need for interventions because of bile collection or biliary peritonitis.^[20]^ BL that required no or little change in a patient's clinical management was considered Grade A leakage, whereas leakage requiring additional diagnostic or interventional procedures was considered Grade B leakage. Grade A leakage lasting longer than 1 week was also classified as Grade B leakage. BL requiring relaparotomy was considered Grade C leakage.

### 2.3 Definition of related surgery

The management of HAE was basically chosen in line with the WHO guidelines on the treatment of echinococcal disease.^[21]^ The classification of hepatic resection was conformed to the Brisbane 2000 terminology.^[22]^ Hepatic parenchymal transection was performed using the crushing clamp method, ultrasonic dissector (CUSA), and guided ultrasonography. According to the cut surface area of the liver after resection and relationship with the hepatic hilum, the patients were divided into the minimal surface area of hepatectomy (MH) group and large surface area of hepatectomy (LH) group. The LH group had resections with involvement of the hepatic hilum or exposure of the Glisson sheath at the cut surface (i.e., right hemihepatectomy, central bisectionectomy, bisegmentectomy of segment 5.6 or 7.8, bisegmentectomy of segment 4 and 8, caudate lobectomy, left hemihepatectomy and caudate lobectomy, or right anterior sectionectomy). The MH group had resections without involvement of the hepatic hilum or exposure of the Glisson sheath at the cut surface (i.e., left hemihepatectomy, simple wedge resection, local excision of the right or left liver, left lateral sectionectomy).

All surgeries were open. The surgical approaches were determined by each individual liver surgeon. Finally, BL was tested by placing a piece of white gauze on the cut surface of the liver to ensure the presence or absence of bile water. If patients underwent hemi-hepatectomy or resection of multiple segments of the liver, BL tests were empirically performed through the cystic duct of the gallbladder.^[12]^ If BL was found, primary sutures, primary sutures with cholecystectomy or t-tube drainage was performed. Next, an absorbable hemostat was used to the cut surface of the hepatectomy. External drainage, omentoplasty, or capitonnage was determined by each surgeon for cavity management. Drainage was often performed using 2 rubber tubes. If there was no abnormal situation, the doctors would usually pull out the drainage tube on the fifth day after surgery.

Postoperative antibiotics were selected according to the general situation of our hospital infections. Usually antibiotics were not administered for more than 3 days after the operation. All patients were administered albendazole (10–15 mg/kg/day) postoperatively for 1 year. Albendazole was stopped if patients showed adverse events (including hypersensitivity, drug-induced liver injury, intolerance, or allergy).

### 2.4 Statistical analysis

All quantitative variables that obeyed normal distribution were revealed as the means ± SD and were compared using the independent Student *t* test. The Mann–Whitney *U* test was used to analyze the suitable variables. The categorical data were expressed as frequencies and compared using the Chi-squared test or Fisher exact test. Receiver operative characteristic curve (ROC) analysis was used to determine the cutoff values for those factors and predictive value of a model. All *P* values ≤0.050 and 2-sided were deemed statistically significant. Univariate logistic regression analyses were performed using patient characteristics and surgery-related variables as independent variables and BL as the dependent outcome. Next, selected variables (*P*-value below .050 in univariate analysis) were entered into the multiple logistic regression analysis to investigate the factors related to BL. ROC curve analysis with calculations of the area under the curve (AUC) was used to determine the ideal score values for those variables that were found to be significant. Statistical analysis was performed using SPSS 22.0 for Windows software (SPSS, Inc., Chicago, IL).

## 3 Results

### 3.1 Patient characteristics

We enrolled 85 male patients (55.9%) and 67 female patients (44.1%), with an age range of 6 to 67 years. Twenty-two patients (22/152, 14.5%) classified into the bile leakage present (BLP) group. The remaining 130 patients showed no BL and were classified into the bile leakage absent group (BLA). The baseline data and surgical data of the 2 groups are compared in Table 1. Compared with the BLA group, the BLP group showed no significant difference in age, sex, BMI, duration of disease, PNM stage, blood cell count, AST, direct bilirubin, mass location, the number of lesions, and the method of biliary tract intervention. Preoperative imaging analysis showed that there were no differences between the 2 groups in HAE that invaded the main trunk of the portal vein, hepatic artery, hepatic hilar bile duct, and hepatic vein. Notably, 3 BL patients underwent PTCD to decrease bilirubin before surgery (3/22, 13.6%, *P* = .010), and 14 underwent partial hepatectomy in the BL absent group. Preoperative liver function was obviously different between the groups. The BLP group had a significantly increased level of ALT, ALP, and LDH (*P* = .007, *P* = .028, and *P* = .015, respectively). The blood loss and surface area of hepatectomy in patients who underwent BL were significantly greater than those in the no BL group (*P* = .045 and *P* = .032, respectively), and the hospital stay was longer in the BL group than in the control group (26.32 ± 14.26 and 13.41 ± 6.34, respectively; *P* < .001).

**Table 1:**
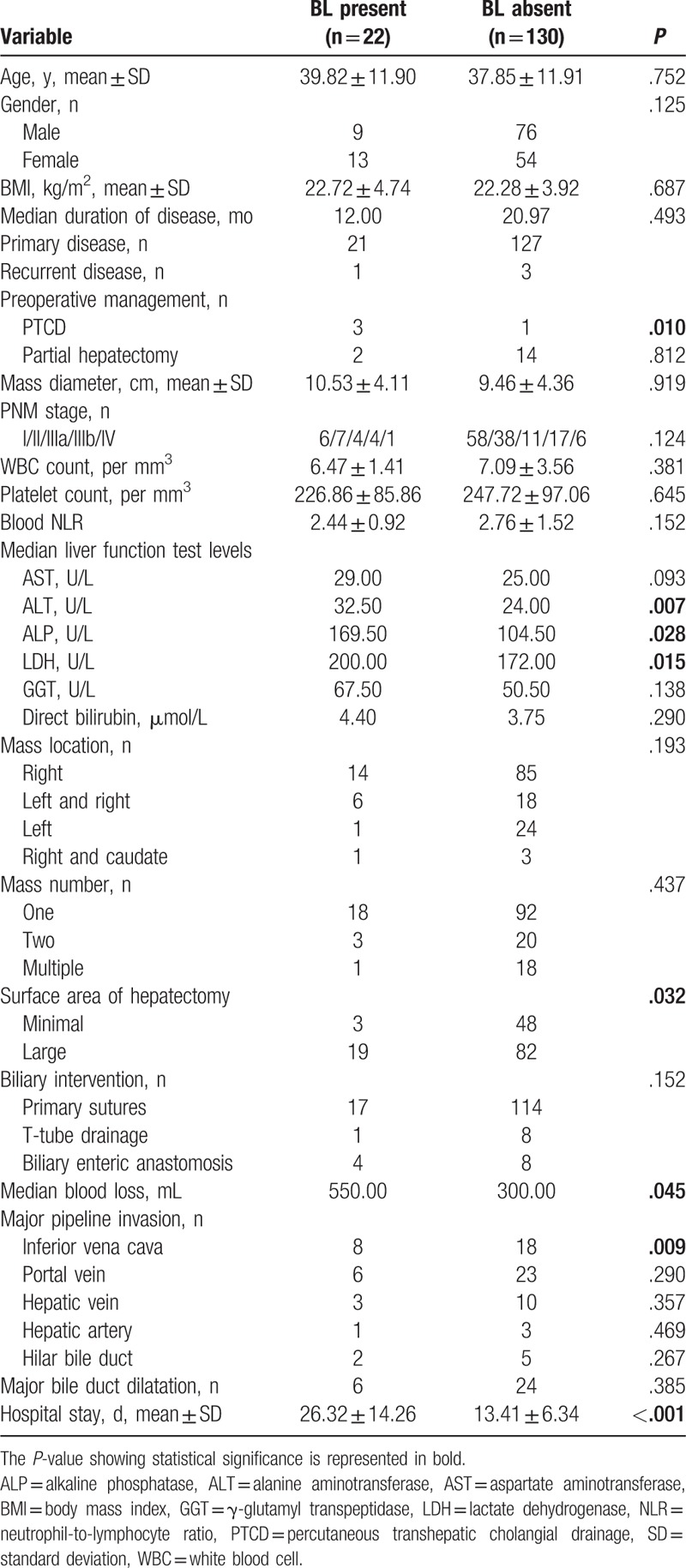
Baseline data and surgical statistics of patients with or without bile leakage (BL).

### 3.2 Postoperative management of the bile leakage present group

Treatment of BL after surgery is still difficult. Twenty-two patients had BL, 13 females and 9 males, with a median age of 41 years. Postoperative BL was found, the earliest on day 3 and the latest on day 10, and the median was postoperative day 5. BL duration ranged from 5 to 34 days. The maximum daily drainage volume (the mixture of bile, ascites, and blood, but bile is the main component) ranged from 80 to 500 mL. Eight patients developed hypokalemia (1/22, 4.5%), pleural effusion (4/22, 18.2%), abdominal abscess (2/22, 9.1%), and acute cholangitis (1/22, 4.5%). Finally, 1 patient underwent relaparotomy (choledochojejunostomy), 7 underwent minimally invasive surgery (endoscopic retrograde cholangiopancreatography (ERCP), PTCD, or abdominal puncture drainage), and 14 retained the drainage tube for continued drainage (63.6%). Based on the consensus proposal of the ISGLS for the grade of BL,^[20]^ 19 patients were classified as Grade B, 2 were classified as Grade A, and 1 was classified as Grade C.

### 3.3 The risk factors for BL detected by univariate and multivariate analyses

**Figure 1:**
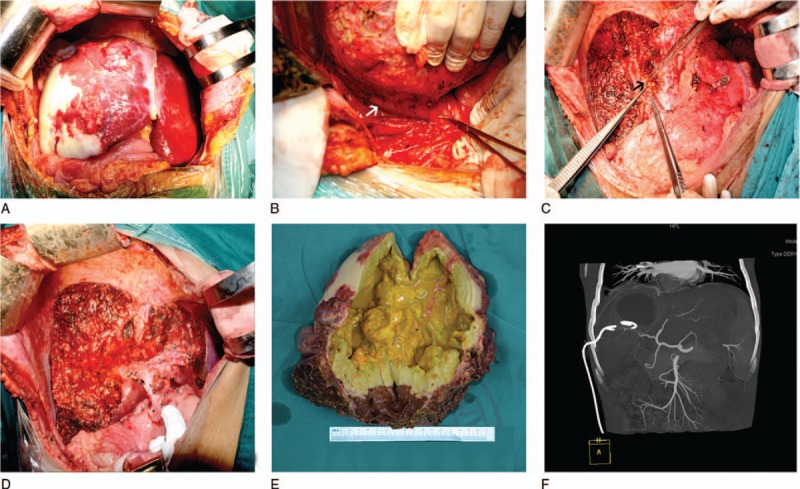
Major risk factors of bile leakage for hepatic alveolar echinococcosis (HAE) in liver surgery and preoperative evaluations. (A) A huge lesion of HAE was approximately 20 cm in diameter in the right liver. (B) An HAE lesion in the right liver involving the inferior vena cava (white arrow). (C) The HAE lesion was removed, and a bile leak (black arrow) was detected under the right portal vein. (D) A larger surface area of liver resection after the HAE lesion was removed. (E) HAE invasion of the liver parenchyma, especially the biliary tract, causing cholestasis (yellow liquid). (F) The drainage tube of PTCD and the lesions are shown on the CT diagram.

Univariate analysis showed that direct bilirubin > 7.1 μmol/L, alanine aminotransferase (ALT) > 28 U/L, ALP > 165 U/L, lactate dehydrogenase (LDH) > 194 U/L, blood loss > 450 mL, mass diameter > 12 cm, inferior vena cava invasion, larger surface area of hepatectomy, and PTCD history were major risk factors for BL (Table 2) (Fig. 1). Statistically significant variables identified by univariate analysis were analyzed by multivariate analysis with the multiple logistic regression analysis to identify the factors associated with BL. The multivariate analyses revealed that direct bilirubin >7.1 μmol/L (OR, 3.565; *P* = .034), LDH > 194 U/L (OR, 4.327; *P* = .005), lesion diameter > 12 cm (OR, 3.043; *P* = .030) and a larger surface area of hepatectomy (OR, 0.198; *P* = .027) were identified as independent risk factors for BL (Table 3).

**Table 2:**
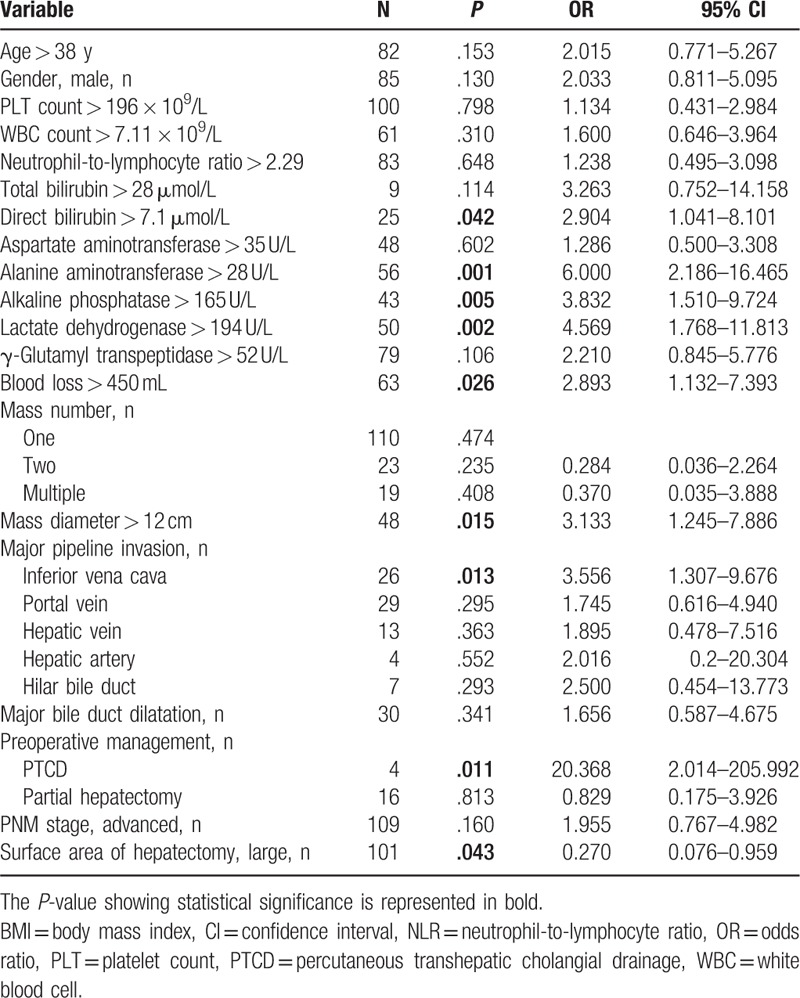
Results of univariate analysis of bile leakage after surgery for HAE.

**Table 3:**
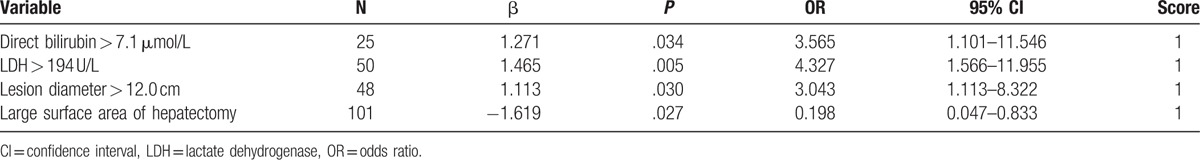
Results of multivariate analyses and associated risk scoring system for bile leakage after operation for HAE.

### 3.4 Establishment and evaluation of a score model

**Figure 2:**
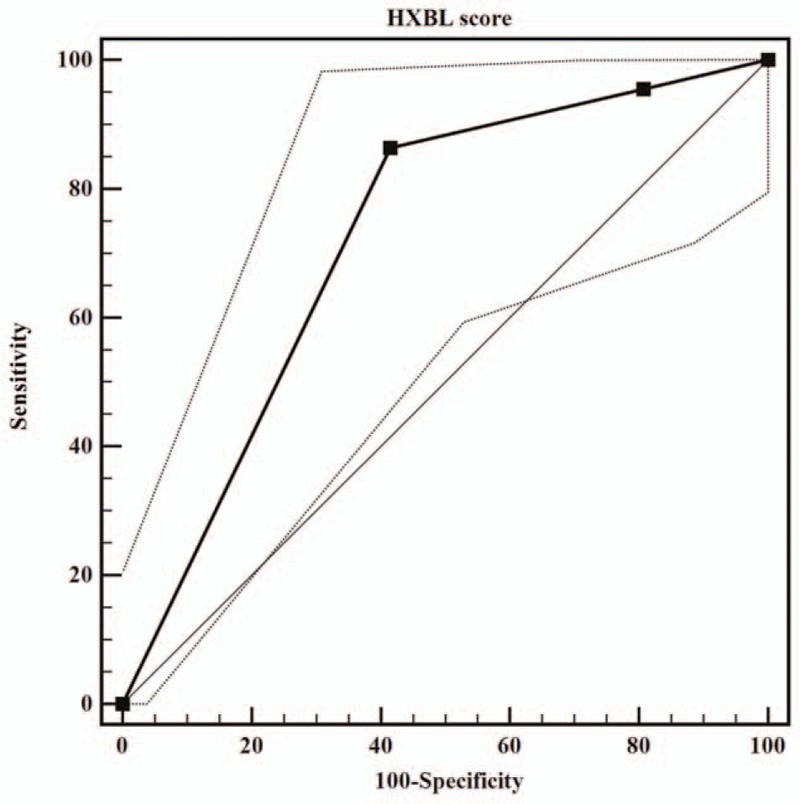
ROC curve analysis of the HXBL score. The resulting area under the ROC of the scoring model was 0.724 (95% CI, 0.646–0.793).

We set up a scoring system and assigned the corresponding numeric hierarchy to some meaningful variables. Expediently, 1 point was assigned to these 4 risk factors (direct bilirubin > 7.1 μmol/L, LDH > 194 U/L, lesion diameter > 12 cm, and a larger surface area of hepatectomy, respectively) (Table 3). The results of scores of HXBL (named after our hospital; bile leakage of Huaxi, Huaxi, China), ranged from I to III, and a total score greater than 2 was defined as III. Based on the HXBL score, all patients were classified into 3 groups (Table 4). For Grade I, the BLP groups accounted for only 4.5%, but 19 patients (86.4%) were listed as grade III in the BLP group. Based on ROC curve analysis of the HXBL score, the sensitivity, specificity, positive predictive value, and negative predictive value for the HXBL score exceeding Grade II were 86.4%, 58.5%, 26.0%, and 96.2%, respectively. The area under the receiver operating characteristic was 0.724 (95% CI, 0.646–0.793) for the HXBL score (Fig. 2).

**Table 4:**
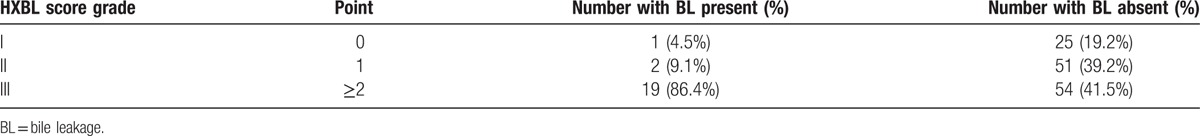
Risk of bile leakage according to the HXBL score.

## 4 Discussion

This study showed that preoperative liver function, surgical intervention, lesion characteristics, blood loss, and the surface area of hepatectomy were the major risk factors for biliary leakage in HAE patients. Hepatic AE is similar to what is called “worm cancer,” which exhibits tumor-like characteristics with liver infiltration, damage of the parenchyma, and invasion of blood vessels or biliary structures.^[19]^ The northwest China and Tibetan regions are the most prevalent areas with HAE.^[23]^ However, because of the early absence of clinical symptoms in HAE patients, most of them are advanced in the treatment of disease. They are characterized by huge masses, impaired liver function and invasion of surrounding organs. Preoperative PTCD and partial hepatectomy give patients who cannot receive timely and effective treatment at the local hospital hope for radical resection and relief of symptoms, especially. However, we found that 3 patients with preoperative PTCD had BL after operation. It may be due to patients with severe biliary obstruction and jaundice before the operation of PTCD.

Preoperative liver function is an important factor in predicting the occurrence of BL. We found that direct bilirubin, ALT, ALP, and LDH were risk factors for BL. Demircan et al^[24]^ reported that direct bilirubin and ALP were risk factors of biliary leakage, a finding that was in line with our research. Notably, the high LDH level (>194 U/L) is an important factor which was found in the study. The liver is one of the major organs that produce LDH which is released into the peripheral blood following liver cell death caused by ischemia or injury. In this study, the LDH level on admission was independently associated with BL in univariate and multivariate analyses. High LDH levels may reflect the number of affected bile ducts and the severity of liver injury by the HAE. Haruki et al^[25]^ found that a lower monocyte count in peripheral blood was an independent risk factor of BL for patients with colorectal liver metastases after hepatic resection on postoperative day 1. A preoperative neutrophil-to-lymphocyte ratio (NLR) ≥2.3 could be a risk factor for surgical complications with colorectal resection in Josse et al^[26]^ study. Unfortunately, our study did not show that the blood count and NLR were associated with postoperative BL.

Preoperative image evaluation can predict the incidence of BL. When the lesion diameter is greater than 12 cm, the rate of BL is higher. Kilic et al^[18]^ suggested that, when the mass diameter was adopted as 7.5 cm, the sensitivity and specificity for biliary-cyst communication were 79% and 73%, respectively. Other authors^[9]^ have found that a lesion size greater than 10.5 cm is a vital predictor of cyst-biliary communication. When HAE masses are large or invade the hepatic portal vessels, various blood vascular complications occur, including the vena–cava syndrome, Budd–Chiari syndrome, and jaundice obstructive or bile duct dilatation due to narrowing of the hepatic veins^[27]^ or inferior vena cava^[28]^ or hilar bile duct, respectively.

The logistic regression analysis showed a high-risk surgical procedure, in which the blood loss and surface area of hepatectomy exposed the hepatic hilum and included the major Glisson sheath as the individual predictor of the occurrence of postoperative BL.^[6,29]^ The greater the cut surface area of hepatectomy, the greater the damage to the small bile ducts. Kajiwara et al^[9]^ set up a risk score model and revealed that the weight of the resected specimen (*P* = .02) and nonanatomical resection (*P* < .001) were independent predictors of postoperative BL. On the other hand, with a greater amount of blood loss, bile duct ischemia may ignore potential bile leak sites for surgeon.^[30]^ Therefore, we suggest that intraoperative blood loss should be avoided, especially when liver resection is performed, and the blood loss volume should be lower than 450 mL.

There are many measures to avoid the occurrence of postoperative BL. Dziri et al^[31]^ reported in a prospective, multicenter, randomized trial that omentoplasty reduces the incidence of BL for hydatid disease of the liver after surgical interventions (pericystectomy or unroofing). However, Paquet et al^[32]^ did not find that omentoplasty could lower the rate of biliary leaks in hepatic resection, and the procedure was not recommended as a routine measure to complete elective hepatic resections. Traditionally, the White Gauze test is a simple and effective technique to stop BL after hepatectomy.^[33]^ These methods were most frequently used at our center, but there was still a relatively higher rate of BL (22/152, 14.5%). Additionally, the intraoperative BL test has been used to detect leakage at several institutions. However, Ijichi et al^[12]^ reported in a randomized controlled research of 103 patients with liver resection that BL tests were not effective to predict the occurrence of BL. At the same time, a prospective cohort study showed^[29]^ that the intraoperative application of an anti-adhesive agent was not related to BL.

We found that BL occurred between postoperative day 3 and 10. Most patients were removed from the peritoneal drainage tube 5 days after surgery. There was a report that drain removal on postoperative day 3 was based on the volume and a safe bile concentration,^[34]^ also suggesting that a drainage volume <102 mL is the only key predictor of spontaneous closure.

In conclusions, our risk score system suggests that direct bilirubin > 7.1 μmol/L, LDH > 194 U/L, a lesion diameter >12 cm and a larger surface area of liver resection were independent risk factors for predicting the risk of BL after hepatectomy. Thus, a high-risk score needs more cautious treatment by an experienced surgeon. It is recommended that radical hepatectomy, not PTCD, be performed as extensively as possible and that the surgeon should learn to avoid bleeding and unnecessary cutting of the liver surface area.
